# Effects of Variations in Color and Organ of Color Expression in Urban Ornamental Bamboo Landscapes on the Physiological and Psychological Responses of College Students

**DOI:** 10.3390/ijerph18031151

**Published:** 2021-01-28

**Authors:** Yuqian Wang, Huahong Qu, Tong Bai, Qibing Chen, Xi Li, Zhenghua Luo, Bingyang Lv, Mingyan Jiang

**Affiliations:** College of Landscape Architecture, Sichuan Agricultural University, Chengdu 611130, China; shewangyq@126.com (Y.W.); quhuahong2020@163.com (H.Q.); baitong315@163.com (T.B.); cqb@sicau.edu.cn (Q.C.); lixi@sicau.edu.cn (X.L.); YLS@sicau.edu.cn (Z.L.); beyonglv@163.com (B.L.)

**Keywords:** urban greening, ornamental plants, color variation, organ expression, physiological and psychological responses

## Abstract

Visual characteristics (e.g., the color and shape) of ornamental plants can significantly affect their beneficial influence on humans. Prior research, however, has largely focused on the effects of the color or shape of flowers and the impact of differences in the visual appearance of foliage plants and plants with ornamental stalks has not yet been fully explored. This study examined the psychophysiological effects of urban ornamental bamboos that expressed different colors on different organs. Three hundred Chinese college students participated in the experiment. They were randomly assigned to view images of five ornamental bamboo landscapes with the following different visual characteristics: green stalks (GS) non-green stalks (NGS) multicolored stalks (MS) green leaves (GL) and multicolored leaves (ML). Before and after viewing the images, their EEG, blood pressure, pulse, profile of mood states (POMS) score, and state-trait anxiety inventory (STAI) score were measured. The results showed that ornamental bamboo landscapes have extremely significant beneficial psychophysiological effects as compared to urban landscapes. After viewing landscapes in the NGS and MS groups, EEG, blood pressure, and pulse rate of subjects showed more beneficial changes. Significant gender differences were observed only in systolic blood pressure and in the vigor score. In addition, an extremely significant interaction between color and organ of color expression was observed on systolic and diastolic blood pressure. Organ of expression had significant main effects on all the physiological indicators and the fatigue, vigor, and irritability scores, while color only had a main effect on systolic blood pressure. Our study concluded that viewing urban ornamental bamboo landscapes with different visual characteristics has different effects on humans. With regards to ornamental bamboo, the organ expressing the color had a greater impact on psychophysiological responses than did the type of color itself. These study results can provide guidance for landscape construction of urban greening.

## 1. Introduction

The global trend of increasing urbanization is coupled to a rise in a range of serious health problems such as diabetes, high blood pressure, and depression. The emergence of various physiological and psychological diseases makes it vital for city dwellers to optimize their urban living environment [1,2]. Studies have proven that the natural environment is more beneficial to the human body than cities in mitigating stress states and inducing physiological relaxation [3,4]. The interaction between human beings and nature can effectively strengthen the human nervous system [5,6], secretory system [7], and immune system [8], while also improving mental health problems such as anxiety and depression [9,10].

The urban natural environment is an important part of the overall urban environment that can potentiate the restorative effects of cities. Appropriate urban natural environment planning is now recognized as an effective means to solve adverse health issues [11,12]. The urban natural environment is composed of different elements, whose combination and layout form a variety of urban landscapes [13,14]. Previous studies have confirmed the restorative effects of different landscape type environments, including lakes, lawns, and mountains [15,16,17]. Rich and diverse vegetation is not only an important component of the urban natural environment but is also an expression of the urban landscape [18,19]. Several studies have shown that different psychophysiological effects such as decreasing blood pressure and relaxing the mind can be caused by the arranging and cultivation of plants [20,21].

Visual information is the primary modality through which people perceive their environment [22]. Plants’ colors have been confirmed to be an important tool for improving public health. The colors and shapes of plants can decrease blood pressure and depressive symptoms and improve positive mood. Several studies have shown that plants with various colors and shapes have benefits for health care, rehabilitation, and emotional regulation [23,24,25]. Color and shape variations are often manifested in plants’ ornamental organs. Recent attention has focused on the physical and mental effects of exposure to flowers with a variety of bright colors [26,27]. As urban greening becomes richer and more colorful, some special ornamental plants whose color variations appear on the leaves and stalks have come into wide use. However, few studies to date have examined whether these foliage plants and plants with ornamental stalks have restorative effects [28].

Bamboo is commonly used for urban landscape greening in Oriental gardens [29]. In recent years, researchers have found that walking in bamboo forests can relieve tension, reduce depression and decrease blood pressure [30,31]. However, in addition to bamboo forests, there are many small bamboo species could be used as ornamental materials in urban greening. They are typical non-flowering ornamental plants and they display very rich yellow-green color variations on the stalks and leaves. Few studies have investigated the effect of this special ornamental plant on environmental quality or people’s physiological and psychological responses. In addition, when this plant is applied in urban greening, the variations in color itself and variations in the organs that express the colors might also exert different influences. However, very few studies have considered how these changes affect human health.

Thus, this study assigned college students to view photographs of ornamental bamboo landscapes and monitored their psychophysiological indicators. From the perspective of the color of stalks and leaves, this study aimed to explore the various effects of urban ornamental bamboo landscapes with different color variation and the organs of color expression, and the findings can be applied to urban greening.

## 2. Materials and Methods

### 2.1. Participants

In this study, posters were placed around a local university to recruit participants from all over the country. Three hundred participants aged from 18 to 28 years (mean age: 22.41 ± 1.96 years; gender proportion: 1:1) enrolled in the experiment. To assure their approval of this study, every participant received detailed information about the experiment. All of the participants were without any disease. Smoking, drinking, coffee drinking, and vigorous exercise were banned before the experiment. The study was conducted with the permission of the local Ethics Committee. The ethical approval number is SICAU201504120023

All of the participants were randomly assorted into five groups consisting of sixty individuals each (gender proportion: 1:1). We chose April 2015 (from 9:00 to 11:00 and 14:00 to 17:00 every experiment day) as experiment times. Ten participants were measured each day and the experiment took 30 days in total. Each group was assigned to view a specific group of ornamental bamboo landscapes. No control group was set up but each group viewed the urban images (control condition) before the green condition. A detailed description of this study was given to each participant to allay any potential anxiety in regard to their participation. All of the participants submitted their written informed consent after confirming that they understood the purpose and design of the experiment.

The experiment was undertaken in a quiet and ventilated room at a local university. The room temperature was maintained at 20 to 24 °C and relative humidity at 40% to 50% to ensure participants’ comfort.

### 2.2. Stimuli

Ornamental bamboos were divided into groups according to their visual characteristics: with regard to stalk color: green stalks (GS) non-green stalks (NGS) and multicolored stalks (MS); with regard to leaf color: green leaves (GL) and multicolored leaves (ML) (we note that commonly used ornamental bamboos do not have non-green leaves).

Twelve species of common ornamental bamboos were chosen to represent each of the five groups, and thus a total of 60 species of bamboo were used to create landscape photos for use as visual stimuli. Arbor-like ornamental bamboos with tall stalks were usually planted in clusters; their stalks were their predominant ornamental organs. Shrub-like ornamental bamboos were low and creeping, usually planted in pieces; their leaves were their predominant ornamental organs. Photos of some species of ornamental bamboo were taken by the same person using the same camera (D90, Nikon Imaging, China) Sales Co., Ltd., Shanghai, China) in Wangjianglou Park, Chengdu, Sichuan, in March 2015. The remaining species of ornamental bamboo, which were too rare for collection of photos, were represented by photos quoted from Iconographia Bambusoidearum Sinicarum [32] and Plant Photo Bank of China (PPBC) [33].

**Figure 1:**
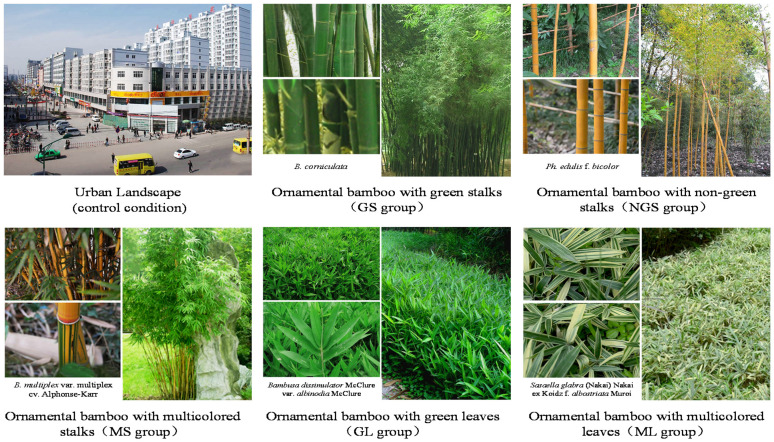
Stimulus images. GS group: ornamental bamboo with only green stalks; NGS group: ornamental bamboo with only non-green stalks (e.g., yellow, white, purple); MS: the stalks of ornamental bamboo appear in two or more colors; GL: ornamental bamboo with only green leaves; ML: the leaves of ornamental bamboo appear in two or more colors.

Peoples’ responses to plants and landscapes can be probed using displayed images. In this study, urban landscape images (control group) and 60 kinds of ornamental bamboo images were made into six PowerPoint files. Each file included 12 slides and each slide was displayed for 10 s. Complete viewing of all slides in a file took 2 min (Figure 1).

### 2.3. Measurements

#### 2.3.1. Physiological Indicators

EEG (electroencephalogram; α and β wave (HZ)) data was collected to reflect the emotional changes in the human body using a portable EEG device (MindSet, NeuroSky Mind Wave Beijing Oriental Creation Technology Co., Ltd., Beijing, China). The increase in high α wave and the decrease in high β brain wave indicated relaxation of the human brain [34,35]. Blood pressure (SBP: systolic blood pressure (mmHg), DBP: diastolic blood pressure (mmHg),) and pulse rate(bpm), reflecting emotional stress were measured with a sphygmomanometer (Omron, HEM-7201, Guangdong, China). The decrease in systolic blood pressure, diastolic blood pressure, and pulse rate indicated reduction in tension [36].

#### 2.3.2. Psychological Indicators

Profile of mood states (POMS) questionnaires were used to assess the psychological responses of participants to ornamental bamboo landscapes. Considering the background of the participants, the questionnaires revised by Morfeld were translated into Mandarin. It was comprised of four scales measured using 24 items. The four scales were irritability, numbness, vigor, and fatigue [37]. Each item was assessed by the participants in a five-point Likert scale, (from “1-not at all” to “5-extremely). The questionnaire was presented in Appendix A (Table A1).

The State trait anxiety inventory (STAI) was used to measure the emotional state of the participants. A questionnaire revised by Spielberg and Gorsuch was translated into Mandarin by Chinese researchers for this study. The Inventory measures 10 positive emotions and 10 negative emotions using a total of 20 questions [38]. Each item was assessed by the participants in a four-point scale, (from “1-not at all” to “4-very much”). The questionnaire was presented in Appendix A (Table A2).

### 2.4. Study Protocol

**Figure 2:**
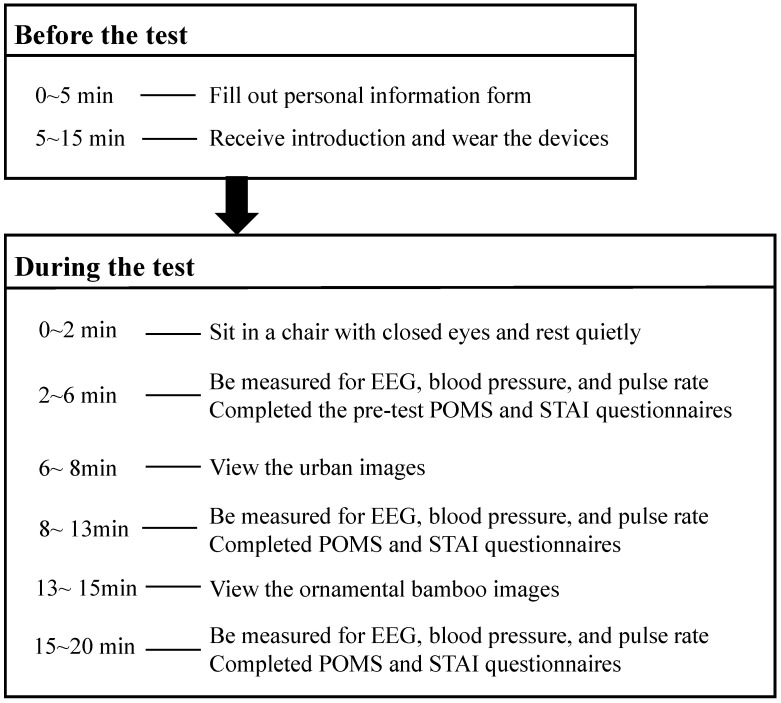
Experimental Design.

Each subject participated in the experiment individually and each experiment took 35 min. As shown in Figure 2, before the experiment, participants recorded their basic personal information in the waiting room and received detailed introductions for the experiment. Other than the fact that different ornamental bamboo landscape images were used, all of the procedures were the same for each participant. There were no order effects in that each participant only viewed one group of images.

After being fitted with the measuring devices, participants entered the experimental room individually. First, the participants’ EEG, blood pressure, and pulse rate were measured in a quiet state and the participants completed the pre-test POMS and STAI questionnaires. Second, the participants viewed the urban landscape images. After viewing the images, participants’ EEG, blood pressure, and pulse rate indicators were measured again. Third, the participants viewed ornamental bamboo landscape images. The same physiological indicators were measured and participants completed the post-test POMS and STAI questionnaires.

### 2.5. Data Analysis

Excel 2016 (Microsoft Corp., Redmond, WA, USA) was used for data statistics and SPSS 20.0 (IBM Corp, Armonk, NY, USA) was used for analysis.

Paired t-tests were used to compare the mean value x_1_ and x_2_ (x_1_ = x_c_ − x_r_, x_2_ = x_b_ − x_r_; x_r_: test values in a quiet state, x_c_: test values after viewing urban images, x_b_: test values after viewing bamboo images) of psychophysiological data (EEG, blood pressure, pulse rate, POMS score, and STAI score) for ornamental bamboo and urban images.

ANOVA (Analysis of variance) was used to analyze the differences in mean restorative value x_3_ of psychophysiological data (x_3_ = x_b_ − x_c_; x_b_: test values after viewing bamboo images, x_c_: test values after viewing urban images) between the five ornamental bamboo groups.

Independent t-tests were used to analyze the differences in mean restorative value (x_3_) of psychophysiological data of different genders.

ANCOVA (Analysis of covariance) was used to calculate the differences in mean restorative value (x_3_) of EEG, blood pressure, POMS, and STAI indicators among the five groups. Color and organ were used as independent variables to conduct an interaction effect analysis and to compare the differences in all psychophysiological indicators under the influence of a single variable. Simple-effects analysis was then conducted on the indicators and a significant interaction effect of color and organ was found.

## 3. Results

### 3.1. Effects of Ornamental Bamboo Landscapes on Psychophysiological Responses

#### 3.1.1. Physiological Effects

**Figure 3:**
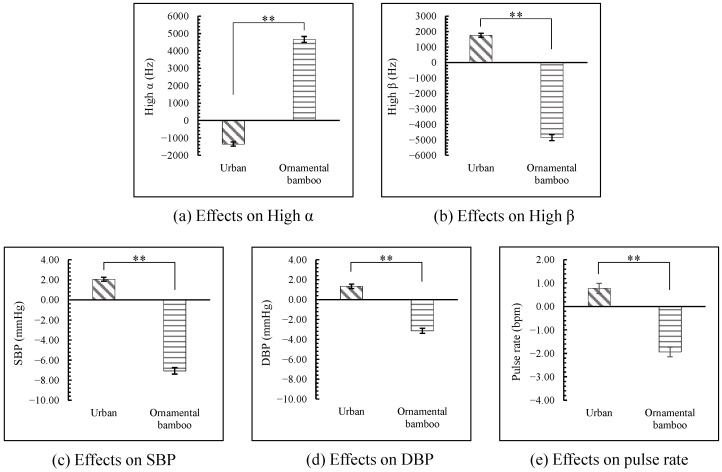
Comparison of physiological indicators after the participants viewed the stimulus images between urban and ornamental bamboo groups. The zero point represents the value measured after participants having a rest. SBP: systolic blood pressure; DBP: diastolic blood pressure; *n* = 300; mean ± SD; ** *p* < 0.01.

All of the physiological indicators showed significant differences (*p* < 0.01) between the ornamental bamboo groups and the urban group after the participants viewed the images. As shown in Figure 3, after the participants viewed the urban images compared to rest, their high α declined and their high β, blood pressure (SBP and DBP), and pulse increased. By contrast, after participants viewed the ornamental bamboo images, their high α increased and their high β, blood pressure, and pulse declined (*p* < 0.01, Figure 3).

#### 3.1.2. Psychological Effects

**Figure 4:**
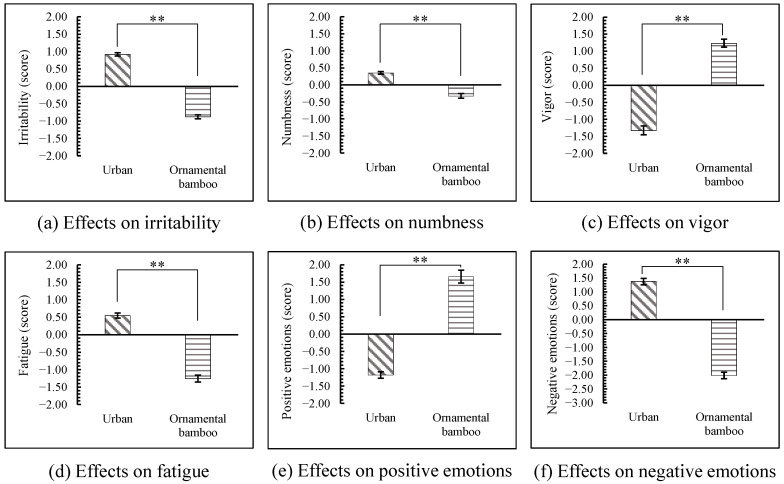
Comparison of psychological indicators after the participants viewed the stimulus images between urban and ornamental bamboo groups. The zero point represents the value measured after participants having a rest. *n* = 300; mean ± SE; ** *p* < 0.01.

Compared with the urban environment, significantly beneficial changes were observed on all psychological responses after the participants viewed the ornamental bamboo images (*p* < 0.01). The POMS results showed that 3 negative emotions including irritability, numbness, and fatigue were significantly lower for the ornamental bamboo groups than for the urban group and 1 positive emotion (vigor) was significantly higher than for the urban group (*p* < 0.01, Figure 4). Similarly, the results from the STAI questionnaires showed that after viewing the ornamental bamboo images, participants’ positive emotions were significantly higher than was observed with the urban group while negative emotions were lower than for the urban group (*p* < 0.01, Figure 4).

### 3.2. Effects of Ornamental Bamboo Landscapes on Psychophysiological Responses

#### 3.2.1. Physiological Effects

All physiological indicators exhibited better values after viewing the NGS and MS groups as compared to the other 3 bamboo groups. None of the differences between the NGS and MS groups were statistically significant (*p* > 0.05).

**Figure 5:**
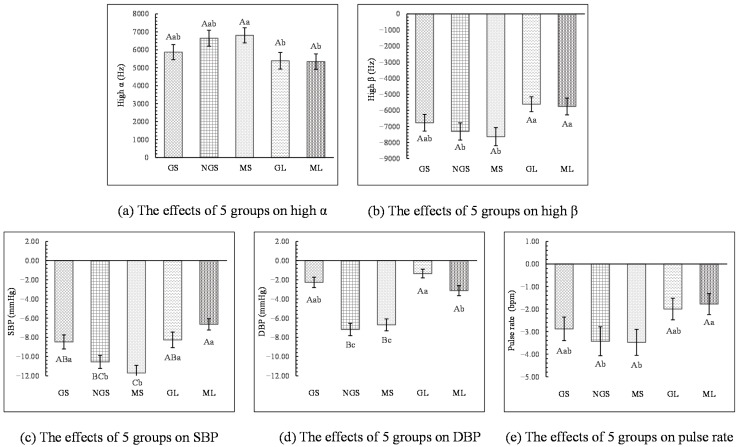
Comparison of the physiological restorative values after the participants viewed five ornamental bamboo groups: GS (green stalk), NGS (non-green stalk), MS (multicolored stalk), GL (green leaf), ML (multicolored leaf). The zero point represents the value measured after participants viewing urban images. SBP: systolic blood pressure; DBP: diastolic blood pressure; *n* = 300; mean ± SE. Different capital letters: significant differences (*p* < 0.01) between five groups. Different lowercase letters: significant differences (*p* < 0.05) between five groups. The same letters: no significant differences.

The EEG results showed that the high α values of the NGS and MS groups (NGS: 6648.53 ± 445.88 Hz; MS: 6805.98 ± 421.26 Hz) were significantly higher than those of the GL and ML groups (*p* < 0.05, Figure 5). Also, the high β values of the NGS and MS groups (NGS: −7315.65 ± 537.59 Hz; MS: −7638.48 ± 567.30 Hz) were significantly lower than those of the GL and ML groups (*p* < 0.05, Figure 5). The high α and high β values did not show any significant differences between the GS, GL, and ML groups (*p* > 0.05).

As shown in Figure 5, significant differences in blood pressure were observed among the 5 groups. The SBP values for the MS group (MS: −11.72 ± 0.80 mmHg) were significantly lower than the values for the GS, GL, and ML groups (*p* < 0.01). The SBP values of the NGS group (−10.57 ± 0.68 mmHg) were significantly lower than those of the GS and GL groups (*p* < 0.05) and significantly lower than those of the ML group (*p* < 0.01). ANOVA found no significant differences in SBP values among the GS, GL, and ML groups (*p* > 0.05, Figure 5). The DBP values of the NGS and MS groups (NGS: −7.18 ± 0.64 mmHg; MS: −6.70 ± 0.63 mmHg) were significantly lower than those of the GS, GL, and ML groups (*p* < 0.01) and the GL group had significantly lower values than the ML group (*p* < 0.05, Figure 5).

The pulse rates of the NGS and MS groups (NGS: 3.43 ± 0.64 bpm; MS: −3.48 ± 0.58 bpm) showed significant differences from the ML group (ML: −1.78 ± 0.47 bpm, *p* < 0.05) but no significant differences from the GS and GL groups (*p* > 0.05). The GS, GL, and ML groups had non-significant differences between each group (*p* > 0.05, Figure 5).

#### 3.2.2. Psychological Effects

**Figure 6:**
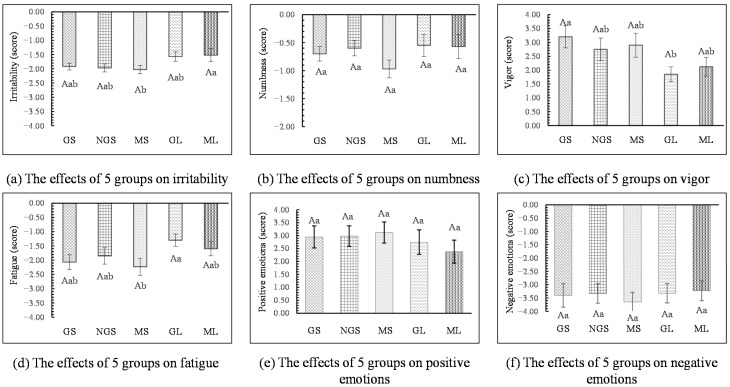
Comparison of the psychological restorative values after the participants viewed five ornamental bamboo groups: GS (green stalk), NGS (non-green stalk), MS (multicolored stalk), GL (green leaf), ML (multicolored leaf). The zero point represents the value measured after participants viewing urban images. *n* = 300; mean ± SE. Different capital letters: significant differences (*p* < 0.01) between five groups. Different lowercase letters: significant differences (*p* < 0.05) between five groups. The same letters: no significant differences.

The mean psychological restorative values after the participants viewed stimulus images are shown in Figure 6. Unlike the physiological indicators, the differences in the five groups were relatively small and the ability to improve emotions was similar among the five groups. In the POMS results, the MS group showed the lowest negative emotion score of all the five groups. The irritability value of the MS group (MS: −2.03 ± 0.15) was significantly lower than that of the ML group (ML: −1.52 ± 0.23, *p* < 0.05, Figure 6) and the fatigue value was significantly lower than that of the GL group (MS: −2.23 ± 0.30; GL: −1.30 ± 0.21, *p* < 0.05, Figure 6). Also, the vigor value of the GS group (GS: 3.20 ± 0.40) was significantly higher than that of the GL group (GL: 1.85 ± 0.27, *p* < 0.05, Figure 6). In addition, there was no significant difference in STAI results (positive emotions and negative emotions) among the five groups (*p* > 0.05, Figure 6).

#### 3.2.3. Gender Difference

As shown in Table 1, after viewing the ornamental bamboo images, the mean restorative value of physiological and psychological indicators of women showed more obvious beneficial changes. However, significant gender differences were observed only in the SBP index of the MS group and the vigor index of the ML group (*p* < 0.05, Table 1).

### 3.3. Interaction Effects of Color and Organ on Psychophysiological Responses

#### 3.3.1. Effects of Color and Organ on Humans

Table 2 showed the results of ANCOVA examining the effect of color (green/other colors) and the organ on which the color was expressed (stalks/leaves) on physiological and psychological responses. A significant interaction was found between the influence of color and organ on all physiological indicators. Organs had significant main effects on high α and pulse indicators (*p* < 0.05), and significant main effects on high β, SBP, and DBP (*p* < 0.01) whereas color had significant main effects only on SBP (*p* < 0.01, Table 2). This result is in line with the preceding results that showed that beneficial changes in physiological indicators were greater after viewing the NGS and MS groups than the ML group.

In addition, the psychological results showed that organs had significant main effects on fatigue (*p* < 0.05) and significant main effects on irritability and vigor (*p* < 0.01). However, no significant main effects of color on psychological indicators were observed.

#### 3.3.2. Interaction Effects of Color and Organ on Physiological Responses

**Figure 7:**
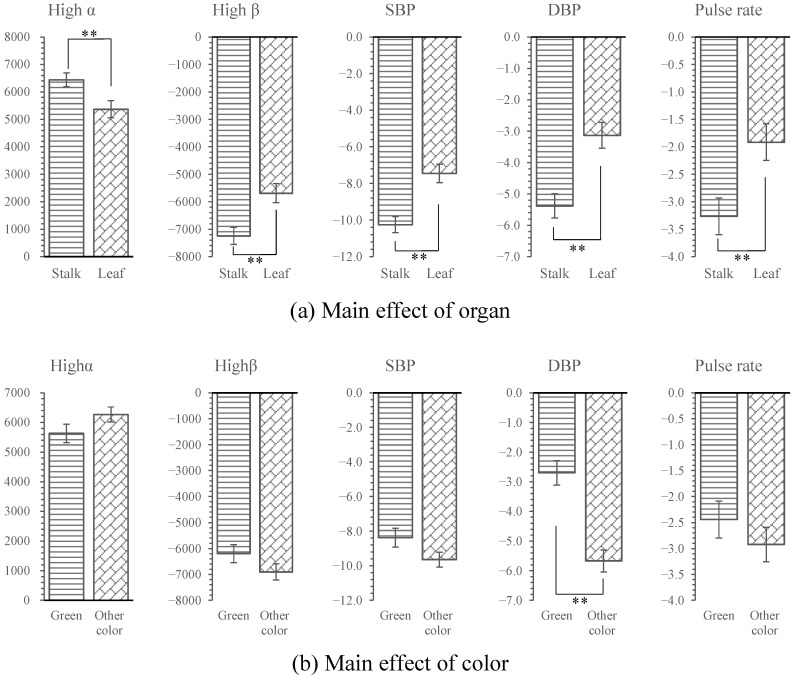
Mean effect of organ and color on physiological indicators. The zero point represents the value measured after participants viewing urban images. SBP: systolic blood pressure; DBP: diastolic blood pressure, *n* = 300; mean ± SE, ** *p* < 0.01.

As shown in Figure 7, based on the main effects of organs, all physiological beneficial effects of the leaf groups (high α: 5367.93 Hz; high β: −5689.37 Hz; SBP: −7.64 mmHg; DBP: −3.13 mmHg; pulse rate: −1.92 bpm) were significantly lower than those of the stalk groups (high α: 6441.94 Hz; high β: −7243.19 Hz; SBP: −10.26 mmHg; DBP: −5.38 mmHg; pulse rate: −3.27 bpm, *p* < 0.01, Figure 7). Based on the main effects of colors, only the DBP of the green group (DBP: −2.70 mmHg) was significantly higher than that of the other colors group (DBP: −5.67 mmHg, *p* < 0.01, Figure 7).

**Figure 8:**
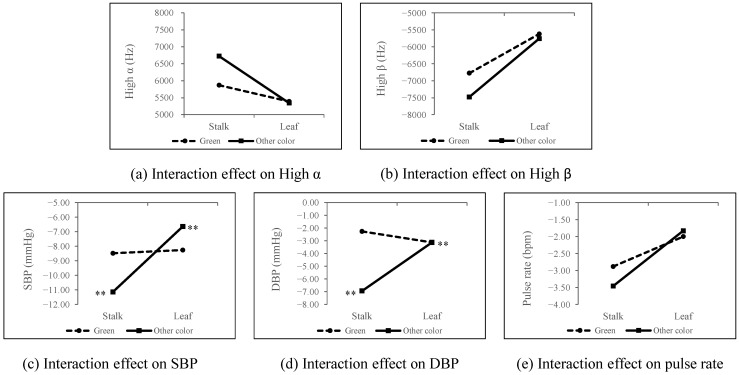
Interaction effect of organ and color on physiological indicators. SBP: systolic blood pressure; DBP: diastolic blood pressure, *n* = 300; mean ± SE, ** *p* < 0.01.

Figure 8 shows the interaction effects of color (green/other colors) and organ (stalks/leaves) on physiological responses. After the participants viewed the ornamental bamboo images, the beneficial changes in high α, high β, and pulse rate for the other colors group were found to be higher than those for the green group. In addition, significant interaction effects of color and organ were observed on SBP and DBP (*p* < 0.01, Figure 8). Simple-effects analysis was further conducted on these two indicators and both indicators for the stalk group [SBP: −11.14 mmHg; DBP: −6.94 mmHg] were found to be significantly lower than those for the leaf group [SBP: −6.65 mmHg; DBP: −3.13 mmHg], (color = “other color”), showing that SBP and DBP were influenced by the interaction effects of color and organ.

#### 3.3.3. Interaction Effects of Color and Organ on Psychological Responses

**Figure 9:**
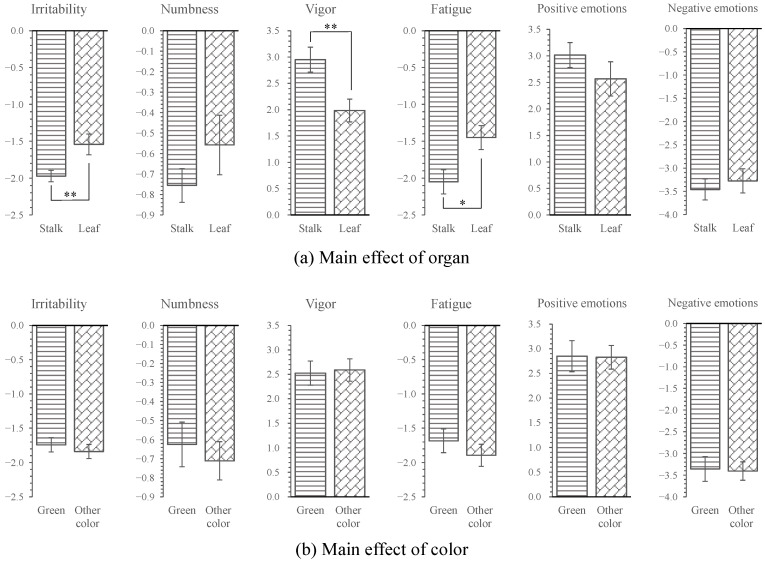
Mean effect of organ and color on psychological indicators. The zero point represents the value measured after participants viewing urban images. *n* = 300; mean ± SE, * *p* < 0.05; ** *p* < 0.01.

As shown in Figure 9, based on the main effects of organs, the beneficial changes to the irritability and vigor indicators in the leaf groups (irritability: −1.54; vigor: 1.98) were significantly lower than those of the stalk groups (irritability: −1.97; vigor: 2.95, *p* < 0.01) and the beneficial changes in fatigue for the leaf groups (fatigue: −1.45) were significantly lower than those for the stalk groups (fatigue: −2.05, *p* < 0.05, Figure 9). Based on the main effects of colors, no significant difference was found for any psychological indicators (*p* > 0.05, Figure 9).

**Figure 10:**
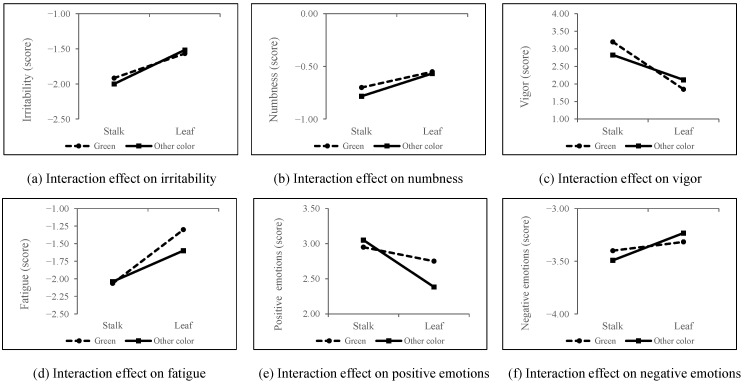
Interaction effect of organ and color on psychological indicators. *n* = 300; mean ± SE.

Figure 10 shows that no significant interaction effects of color and organ were found for psychological responses (*p* > 0.05). Psychological indicators did not vary based on color and organ.

## 4. Discussion

### 4.1. Beneficial Effects of Foliage Plants and Plants with Ornamental Stalks on Humans

This study selected typical Chinese urban greening plants as experimental materials. From the perspective of color variation and organ expression, it was found that various ornamental bamboos with different visual characteristics can have significantly beneficial effects on the human body. Compared with urban environment, ornamental bamboos can change EEG, reduce blood pressure and pulse rate, also, alleviate negative mood. Thus, we thought that ornamental bamboos might mitigate the diseases related to blood pressure, anxiety, and depression, making people feel comfortable and relaxed. This result is consistent with previous studies [39]. Several previous studies have shown that contact with urban natural landscapes brings a range of health benefits. Jeon et al. found that exposure to natural environment appears to be beneficial to participants’ moods and feelings [40]. Mao et al. demonstrated that forests have therapeutic effects on human hypertension and can inspire its preventive efficacy against cardiovascular disorders [41]. When people either view or are present in the natural environment, various physiological indicators (heart rate, blood pressure, EEG, etc.) and psychological indicators (anxiety, depression, attention, etc.) show significant beneficial changes [42,43]. In addition, when people appreciate the plants, the first thing they see is the color, which strongly affects their response to the plants [44]. Some studies have found that flowers of different flowering periods or different colors have a range of influences on psychological responses [45,46,47]. In urban greening, the main ornamental organs of many plants are their branches, trunks, and leaves. Current studies showed that even trees without leaves in the winter season have significantly beneficial psychological effects [48].

This study also found that non-green and multicolor stalks and leaves can have more beneficial physiological effects than green stalks and leaves, and can thus reduce feeling of tension, anxiety, and depression. This result is contrary to several previous studies. Some studies on indoor ornamental plants found that when people observed a green plant, their EEG was more active than when viewing yellow, pink, or red plants [49]. Also, whether the comparison is between plants of the same or different species, green leaves can promote more beneficial effects on EEG than leaves of other colors [50]. The differences in these results may be due to the different perceptions of indoor and outdoor ornamental plants. Further research is needed to confirm the findings of this study.

In terms of psychological effects, this study found that other colored (i.e., non-green) stalks and leaves have a better influence on humans. This result corresponds with the findings of previous studies on the psychological effects of different tree species [27]. In addition, Paraskevopoulou et al. conducted an experiment on the seasonal color changes of plants and argued that patients with psychotic disorders showed more positive facial expressions when viewing an image of a tree with autumn color (yellow) compared to the green one [51]. In this study, we only selected ornamental bamboos with color variations of green, yellow, white, and dark purple as experimental materials, but no bright red or blue. By comparison, these less visually attractive colors may be more in line with people’s preconceptions of plant colors, leading to smaller differences between the psychological responses [52]. Further studies are needed to expand the range of color variation and to confirm the psychophysiological impacts of differently colored plants with non-flower and non-fruit ornamental organs.

### 4.2. Effects of Color Variation and Organ Expression of Plants on Humans

Based on the interaction effect analysis of two variables (color variation and organ expression), this study found interesting results regarding the psychophysiological effects of plant color. In this experiment, significant main effects from color-displaying organs were obtained for all physiological indicators (EEG, blood pressure, and pulse rate.) This result indicates that ornamental bamboo influences humans in ways that depend on the organ which expresses the color. This finding may explain the different results found in previous studies. Previous studies have focused on the effects caused by the color variation of whole plants or a certain organ, while overlooking the potential significance of the type of organ that expresses the color. No studies have yet found a difference in the effects of the expression of color on different organs. However, comparing two experiments on the color of flowers and leaves conducted by Jang, we found that red leaves have more beneficial effects on high β than red flowers do [49,53]. Hůla et al. claimed that the flower color had only a minor effect on peoples’ preference when compared with flower shape [54]. This finding is in line with the results of our study: the organ which expresses color on ornamental bamboo was found to have a greater impact on the psychophysiological response than did differences in color itself.

Significant main effects of the organ expressing color were observed on the psychological parameters of irritability, vigor, and fatigue, indicating that color variation on stalks can bring more beneficial effects than on leaves. This finding is consistent with previous research. Guo et al. reported that *Ginkgo biloba* and *Platanus acerifolia*, both of which have golden yellow leaves, showed significant differences in stress recovery whereas *Ginkgo biloba* and *Sophora japonica*, which both have gray-brown bark but different leaf colors, showed similar ability to decrease stress. However, that study did not consider the possibility of psychological effects caused by different colors on the trunk [55]. In addition, Kuper found that people prefer flowering plants or red, yellow, and autumn-colored foliage, which also have increased psychophysiological effects [56], while Kaufman and Lohr demonstrated that people prefer trees with green and red leaves to those with orange-brown leaves [52]. The findings of this study may explain the differences between the results of Kuper and Kaufman. Furthermore, several previous studies on tree preference found that people generally prefer trees with green leaves [57,58], because green leaves mean healthy plants and a good environment [59]. However, Kendal et al. illustrated that plants with gray and green leaves are equally preferred [60]. The results of these studies agree with our findings: the color of ornamental bamboos has less influence on human psychology or preference than the particular organ which expresses the color. Different ornamental organs change the shape of ornamental bamboos and people may be more affected by these different shapes than by the specific colors.

### 4.3. Limitations and Future Research

There were a number of limitations to this study. First, only typical Chinese ornamental bamboos were selected as the experimental material and the experiment was conducted only on their color variation and organs of color expression. Generalizability may thus be limited. Further research should use other experimental plants and a broader range of colors to obtain more broadly applicable conclusions. Second, the stimulation time of images in this study was relatively short. Real plant materials can be used and the stimulation time can be extended in the future. Third, the background of participants may have confounded the results. Information about what fields the participants majored in and whether they had ever viewed ornamental bamboo landscapes was unknown. Future research should evaluate the physiological and psychological responses according to the different background factors. Forth, the ornamental organs of bamboo are thin and long. It is necessary to carry out further research to confirm whether the results of this study apply to plants with thick tree trunks and round leaves. Fifth, the expression of color in different ornamental organs of different sizes may result in disproportionate displays of color. This study did not consider the impact of color proportion on psychophysiological responses, and this potential effect should be explored in future research. Sixth, the lack of interaction effects found in this study may be due to the relatively small sample size. A large sample are needed on further studies.

## 5. Conclusions

The results of our research showed that viewing urban ornamental bamboo landscapes with different visual characteristics has different psychophysiological effects on humans. Ornamental bamboo with non-green stalks or multicolored stalks can affect humans more beneficially than bamboo with green stalks and can thus reduce feelings of tension and anxiety. We can use more ornamental bamboo with rich color variations on the stalks, so as to promote the feelings of relaxation when people viewing the bamboo landscape.

With regards to ornamental bamboo, a plant with slight variations in color, the organ expressing the color had a greater impact on psychophysiological responses than did the type of color itself. We suggested that a rich variety of plants is not the only goal of urban greening. When arranging non-flower and non-fruit ornamental plants with slight color variations, one can choose plants with varying colors of ornamental organs such as tree trunks, branches, or stems and more creatively alter the expression of colors on these organs to enhance their beneficial effects.

## Figures and Tables

**Table 1:** Mean restorative value of psychophysiological responses of different genders for the five ornamental bamboo groups.

Indicators	Gender	GS	NGS	MS	GL	ML
High α (Hz)	Male	5632.93 ± 604.486	6241.97 ± 565.896	6426.67 ± 493.354	5179.87 ± 588.473	5162.77 ± 565.238
	Female	6109.7 ± 592.569	7055.1 ± 690.868	7185.3 ± 684.657	5599.97 ± 716.54	5529.1 ± 654.611
High β (Hz)	Male	−6999.47 ± 770.15	−7189.5 ± 774.992	−7217.93 ± 759.6	−5478.57 ± 646.115	−5094.63 ± 711.227
	Female	−6551.4 ± 698.177	−7441.8 ± 757.789	−8059.03 ± 848.712	−5762.53 ± 673.193	−6421.73 ± 755.305
SBP (mmHg)	Male	−7.4 ± 1.127	−9.57 ± 1.038	−10.1 ± 1.051 *	−7.8 ± 1.252	−6.8 ± 0.694
	Female	−9.57 ± 0.916	−11.57 ± 0.857	−13.33 ± 1.141 *	−8.73 ± 1.03	−6.5 ± 0.95
DBP (mmHg)	Male	−2.23 ± 0.706	−7 ± 0.788	−6.17 ± 0.708	−0.9 ± 0.485	−2.77 ± 0.845
	Female	−2.3 ± 0.832	−7.37 ± 1.027	−7.23 ± 1.041	−1.8 ± 0.762	−3.5 ± 0.655
Pulse rate (bpm)	Male	−2.13 ± 0.789	−2.37 ± 0.777	−2.9 ± 0.695	−1.53 ± 0.688	−1.37 ± 0.722
	Female	−3.63 ± 0.677	−4.5 ± 0.994	−4.07 ± 0.92	−2.47 ± 0.664	−2.2 ± 0.59
Irritability	Male	−1.8 ± 0.155	−1.87 ± 0.208	−1.9 ± 0.211	−1.33 ± 0.221	−1.23 ± 0.233
	Female	−2.03 ± 0.182	−2.07 ± 0.197	−2.17 ± 0.209	−1.8 ± 0.246	−1.8 ± 0.388
Numbness	Male	−0.63 ± 0.189	−0.6 ± 0.156	−0.9 ± 0.216	−0.63 ± 0.323	−0.57 ± 0.341
	Female	−0.77 ± 0.177	−0.6 ± 0.228	−1.03 ± 0.232	−0.47 ± 0.229	−0.57 ± 0.274
Vigor	Male	3 ± 0.575	2.5 ± 0.559	2.57 ± 0.644	1.83 ± 0.375	1.43 ± 0.439 *
	Female	3.4 ± 0.552	3 ± 0.595	3.23 ± 0.587	1.87 ± 0.389	2.8 ± 0.492 *
Fatigue	Male	−1.97 ± 0.373	−1.5 ± 0.291	−2 ± 0.415	−0.97 ± 0.277	−1.7 ± 0.375
	Female	−2.17 ± 0.378	−2.2 ± 0.492	−2.47 ± 0.436	−1.63 ± 0.313	−1.5 ± 0.324
Positive emotions	Male	3.2 ± 0.535	3.23 ± 0.518	3.37 ± 0.456	2.4 ± 0.774	1.97 ± 0.682
	Female	2.7 ± 0.67	2.73 ± 0.601	2.87 ± 0.679	3.1 ± 0.541	2.8 ± 0.578
Negative emotions	Male	−3.23 ± 0.623	−3.33 ± 0.584	−3.37 ± 0.492	−3.13 ± 0.348	−2.9 ± 0.446
	Female	−3.57 ± 0.637	−3.33 ± 0.451	−3.93 ± 0.54	−3.5 ± 0.641	−3.57 ± 0.598

Note: GS: green stalk, NGS: non-green stalk, MS: multicolored stalk, GL: green leaf, ML: multicolored leaf. *n* = 300; mean ± SE * *p* < 0.05.

**Table 2:** Main effect and interaction effect of colors and organs on the psychophysiological indicators.

Psychophysiological Indicators		df	F	*p*	η_p_^2^
High α	Organ	1	5.235	0.023 *	0.017
	Color	1	0.995	0.319	0.003
	O × C	1	1.222	0.270	0.004
High β	Organ	1	8.676	0.003 **	0.028
	Color	1	0.740	0.390	0.002
	O × C	1	0.334	0.564	0.001
SBP	Organ	1	12.048	0.001 **	0.039
	Color	1	0.590	0.443	0.002
	O × C	1	9.932	0.002 **	0.032
DBP	Organ	1	7.012	0.009 **	0.023
	Color	1	17.710	0.000 **	0.056
	O × C	1	17.710	0.000 **	0.056
Pulse rate	Organ	1	6.138	0.014 *	0.020
	Color	1	0.160	0.689	0.001
	O × C	1	0.540	0.463	0.002
Irritability	Organ	1	7.306	0.007 **	0.024
	Color	1	0.012	0.914	0.000
	O × C	1	0.187	0.666	0.001
Numbness	Organ	1	1.311	0.253	0.004
	Color	1	0.097	0.755	0.000
	O × C	1	0.043	0.835	0.000
Vigor	Organ	1	8.715	0.003 **	0.029
	Color	1	0.024	0.877	0.000
	O × C	1	0.847	0.358	0.003
Fatigue	Organ	1	6.006	0.015 *	0.020
	Color	1	0.311	0.577	0.001
	O × C	1	0.435	0.510	0.001
Positive emotions	Organ	1	1.166	0.281	0.004
	Color	1	0.110	0.740	0.000
	O × C	1	0.338	0.561	0.001
Negative emotions	Organ	1	0.228	0.633	0.001
	Color	1	0.000	0.991	0.000
	O × C	1	0.060	0.807	0.000

Note: SBP: systolic blood pressure; DBP: diastolic blood pressure, * *p* < 0.05; ** *p* < 0.01.

## Data Availability

Publicly available datasets were analyzed in this study. This data can be found here: http://www.who.int/mediacentre/factsheets/fs220/en/; http://ppbc.iplant.cn/; http://www.egms.de/en/journals/psm/2007-4/psm000038.shtml.

## Appendix A

**Table A1:** Profile of Mood States (POMS) questionnaire; Profile of Mood States (POMS) questionnaire.

	Not at All	A Little Bit	Moderately	Quite a Bit	Extremely
Lively					
Furious					
Worn out					
Full of pep					
Bad-tempered					
Slowed					
Alert					
Annoyed					
Cheerful					
Weary					
Vigorous					
Spiteful					
Sluggish					
Peeved					
Energetic					
Exhausted					
Dazed					
Fatigued					
Chippy					
Listless					
Angry					
Active					
Nervous					

**Table A2:** State-Trait Anxiety Inventory (STAI) questionnaire.

	Not at All	Somewhat	Moderately	Very Much
I am relaxed				
I feel secure				
I am regretful				
I am tense				
I feel rested				
I feel upset				
I am anxious				
I feel unfortunate				
I am comfortable				
I feel at ease				
I am self-confident				
I am nervous				
I am jittery				
I am high-strung				
I feel content				
I am worried				
I am over-excited				
I am joyful				
I feel calm				
I feel pleasant				
